# Commentary: Point Prevalence and Associated Factors of Hip Displacement in Pediatric Patients With Mitochondrial Disease

**DOI:** 10.3389/fped.2022.894611

**Published:** 2022-06-21

**Authors:** Josef Finsterer

**Affiliations:** Neurology & Neurophysiology Center, Vienna, Austria

**Keywords:** MELAS plus β-ureidopropionase deficiency, lactic acidosis, stroke-like episode, m.3243A>G, mtDNA

We read with interest the article by Shu et al. about an 8-year-old boy with double trouble mitochondrial encephalopathy, lactic acidosis, and stroke-like episodes (MELAS) syndrome due to the mtDNA variant m.3243A>G in *MT-TL1* with a heteroplasmy rate of 65% and β-ureidopropionase deficiency due to the homozygous variant c.977G>A in *UPB1* (1). MELAS phenotypically manifested with developmental delay, epilepsy, a stroke-like lesion (SLL), lactic acidosis, exercise intolerance, myopathy, basal ganglia calcification, hearing loss, and cognitive decline (1). β-ureidopropionase deficiency only manifested with increased amino acids in urine (1). The study is appealing but raises concerns that need to be discussed.

The study lacks the description of diffusion-weighted imaging (DWI), apparent diffusion coefficient (ADC), perfusion-weighted imaging (PWI), and oxygen extraction fraction (OEF) magnetic resonance imaging (MRI). Stroke-like lesions (SLLs), the hallmark of MELAS, are characterized by hyperintense DWI and PWI, and hypointense OEF. ADC maps can be highly variable (2).

We do not agree that the family history for genetic or metabolic disorders was negative (1). The mother of the index patient was of short stature (146 cm) and was carrying the mtDNA variant m.3243A>G with a heteroplasmy rate of 17% (1). According to this constellation, the mother also had a mild MELAS syndrome. We should know if the variant m.3243A>G also manifested with other features in the mother.

What do the authors mean by “level of heterogeneity”? Do they mean “heteroplasmy”? Regarding heteroplasmy, it is crucial to know in which tissue heteroplasmy was determined and whether only a single tissue or multiple tissues were examined for heteroplasmy rates.

There is a discrepancy between the description of the cerebral MRI in the text and the caption of Figure 2. In the main text, the MRI is described as “showing no specific findings” but Figure 2 shows an SLL in the right parieto-occipital distribution (1).

Ptosis as a manifestation of a seizure is quite uncommon (1). We should know if a video is available of those seizures manifesting with ptosis. It should also be clarified whether ptosis was also present without seizures. Although ptosis is a rare phenotypic feature of MELAS, it has been reported occasionally (3, 4), particularly in MELAS patients with myopathy (5).

The study lacks the treatment the patient received for the SLL at the age of 7 years. Was L-arginine administered intravenously? Has the patient received anti-seizure drugs (ASDs) before the age of 7 years? Has the patient had seizures before?

What do the authors mean by “widened bilateral cerebellar hemispheres” (1)? Did the patient have cerebellar atrophy? What were the clinical manifestations of cerebellar atrophy?

Overall, the interesting study has some limitations and inconsistencies that call the results and their interpretation into question. Clarifying these weaknesses would strengthen the conclusions and could improve the status of the study.

## Author Contributions

The author confirms being the sole contributor of this work and has approved it for publication.

## Conflict of Interest

The author declares that the research was conducted in the absence of any commercial or financial relationships that could be construed as a potential conflict of interest.

## Publisher's Note

All claims expressed in this article are solely those of the authors and do not necessarily represent those of their affiliated organizations, or those of the publisher, the editors and the reviewers. Any product that may be evaluated in this article, or claim that may be made by its manufacturer, is not guaranteed or endorsed by the publisher.
